# Sensory adaptation mediates efficient and unambiguous encoding of natural stimuli by vestibular thalamocortical pathways

**DOI:** 10.1038/s41467-022-30348-x

**Published:** 2022-05-12

**Authors:** Jerome Carriot, Graham McAllister, Hamed Hooshangnejad, Isabelle Mackrous, Kathleen E. Cullen, Maurice J. Chacron

**Affiliations:** 1 Department of Physiology, McGill University, Montréal, Canada; 2 Department of Biomedical Engineering, Johns Hopkins University, Baltimore, USA; 3 Department of Otolaryngology-Head and Neck Surgery, Johns Hopkins University School of Medicine, Baltimore, USA; 4 Department of Neuroscience, Johns Hopkins University School of Medicine, Baltimore, USA; 5 Kavli Neuroscience Discovery Institute, Johns Hopkins University, Baltimore, USA

**Keywords:** Neural circuits, Sensory processing

## Abstract

Sensory systems must continuously adapt to optimally encode stimuli encountered within the natural environment. The prevailing view is that such optimal coding comes at the cost of increased ambiguity, yet to date, prior studies have focused on artificial stimuli. Accordingly, here we investigated whether such a trade-off between optimality and ambiguity exists in the encoding of natural stimuli in the vestibular system. We recorded vestibular nuclei and their target vestibular thalamocortical neurons during naturalistic and artificial self-motion stimulation. Surprisingly, we found no trade-off between optimality and ambiguity. Using computational methods, we demonstrate that thalamocortical neural adaptation in the form of contrast gain control actually reduces coding ambiguity without compromising the optimality of coding under naturalistic but not artificial stimulation. Thus, taken together, our results challenge the common wisdom that adaptation leads to ambiguity and instead suggest an essential role in underlying unambiguous optimized encoding of natural stimuli.

## Abstract (web summary)

Sensory adaptation is widely considered to involve a trade-off between optimality and ambiguity. Here, the authors explored sensory adaptation to both naturalistic and artificial stimuli and found that, owing to contrast gain control, there is unambiguous optimized encoding of naturalistic stimuli.

## Introduction

Stimuli experienced within the natural environment typically range over many orders of magnitude^1–6^. Because of inherent physical limits on the firing activities of neurons, sensory pathways must dynamically update their strategies (i.e., adapt) for coding to remain optimized over the natural range^7–16^. Specifically, optimized coding can be achieved by ensuring that the neural tuning curve matches current stimulus statistics in order to maximize information transmission^17–26^ (see^12^ for review). A prominent form of adaptation which has been observed across systems and species is contrast gain control, by which the neural sensitivity decreases as a function of increasing stimulus amplitude such that the neural firing rate remains within the dynamic range in order to optimize coding^17–31^. However, the prevailing view is that the functional advantages of such adaptation occur at the expense of increased coding ambiguity^23,32,33^. This is because a given neural response is elicited by different stimuli depending on context, which poses challenges for correctly interpreting the current stimulus.

Importantly, a critical limitation of prior studies is that they have focused on artificial stimuli (e.g., noise, steps) to demonstrate that adaptation gives rise to coding ambiguity. Thus, whether ambiguity limits the fidelity of information transmission during natural stimulation remains unanswered. Here, to address this question, we focused on how natural self-motion stimuli are encoded by the vestibular system. This essential system detects and transmits information about the detailed timecourse of head motion and contributes to a wide range of functions ranging from reflexes to the highest levels of voluntary behavior^34^. The vestibular system is particularly advantageous in this context because natural self-motion stimuli have been well-characterized^5,35^ and can be precisely mimicked under laboratory conditions^36,37^. Head motion is sensed by the vestibular end organs from which peripheral afferents project to the vestibular nuclei, which in turn target the ventro-posterior lateral (VPL) area of the thalamus^38–40^. Vestibular thalamocortical neurons within VPL project to cortical areas and thus play an essential role in ensuring the accurate coordination of perception and action (reviewed in:^41^). While previous studies have shown that vestibular thalamocortical but not vestibular nuclei neurons display contrast gain control adaptation^40,42^ (reviewed in^34^), the implications of such adaptation on the encoding of natural self-motion stimuli remain unknown to date.

Accordingly, here we recorded from individual vestibular nuclei and their target vestibular thalamocortical neurons during naturalistic stimulation as well as artificial sinusoidal stimulation for comparison. Responses to artificial stimuli were characterized by high-pass tuning and marked phase leads with respect to head velocity, leading to coding ambiguity as multiples values of the instantaneous head velocity stimulus gave rise to the same output firing rate. Remarkably, a fundamental change in response dynamics was observed for vestibular thalamocortical neurons when naturalistic stimuli were presented. Specifically, tuning became more broadband with no significant phase lead across frequencies, indicating that the firing rate response encoded the instantaneous head velocity stimulus with significantly less ambiguity. This change in coding strategy was not trivially inherited from vestibular nuclei neurons or due to differences in stimulus amplitude between both stimulation conditions. Using computational approaches, we show that contrast gain control adaptation can account for this experimentally observed change in coding strategy. Finally, we establish that vestibular thalamocortical neurons more optimally encode naturalistic stimuli than vestibular nuclei neurons irrespective of how self-motion is represented. Taken together, our results suggest that an important function for contrast gain control adaptation is to enable unambiguous optimized encoding of natural stimuli.

## Results

**Fig. 1:**
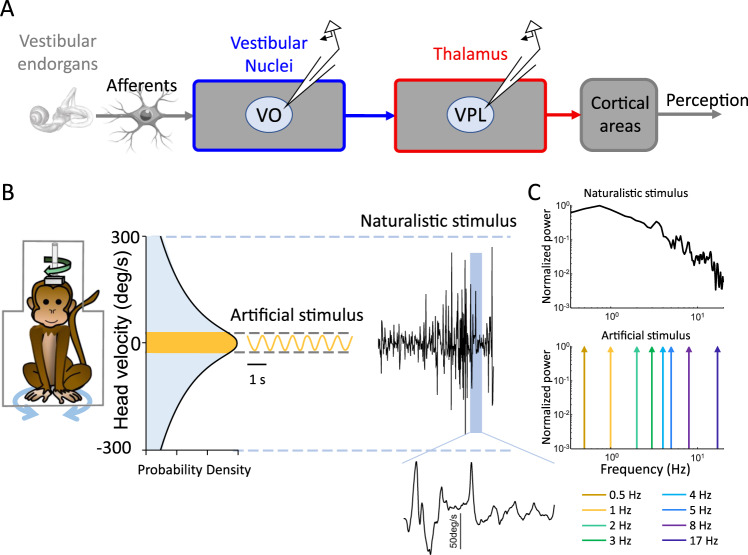
Naturalistic self-motion stimuli strongly differ from artificial self-motion stimuli. **A** Schematic showing vestibular pathways. Self-motion stimuli are transduced by vestibular endorgans and afferents (left) that synapse onto vestibular nuclei neurons (blue). Vestibular-only (VO) neurons within the vestibular nuclei (blue) project to vestibular thalamocortical neurons within the ventral posterior lateral (VPL) area of the thalamus (red). Vestibular thalamocortical neurons in turn project to cortical areas (right) that mediate self-motion perception. **B** Self-motion stimuli were presented using a turntable with the animal headfixed and comfortably seated in a primate chair (left). Natural and artificial self-motion stimuli differ in their characteristics. Most notably, natural self-motion stimuli reach much higher values than artificial ones (right). **C** *Top:* Power spectrum of a natural self-motion stimuli. *Bottom:* Power spectra of all artificial sinusoidal self-motion stimuli used in this study with frequencies spanning the natural range (0.5–17 Hz).

The goal of this study was to investigate how adaptation affects the coding of natural stimuli in the vestibular system. To do so, we recorded the activities of vestibular sensitive neurons within area VPL of the thalamus (i.e., vestibular thalamocortical neurons) projecting to higher brain regions that mediate self-motion perception (e.g., cortex) as well as their input vestibular-only (VO) neurons within the vestibular nuclei (Fig. 1A). Our dataset is comprised of neurons recorded from three awake behaving macaque monkeys (*Macaca mulatta;* VPL: 12 from monkey B, 13 from monkey D, and 3 from monkey S; VO: 14 from monkey B and 13 from monkey D) and for which we were able to maintain isolation during the highly dynamic self-motion stimuli described below. Self-motion stimuli consisted of rotations whose timecourse closely mimicked that recorded while the animal performed natural behaviors (e.g., walking, jumping)^36,37^. These were termed naturalistic self-motion stimuli (see Methods and Fig. 1B). In addition, we applied artificial sinusoidal rotational stimuli that have been typically used to characterize vestibular neural responses whose frequencies spanned the natural range (0.5–17 Hz; Fig. 1B, C). Unlike artificial sinusoidal stimuli which, by definition, each only contain one frequency, naturalistic stimuli contain a spectrum of frequencies and span a wider range of amplitudes (Fig. 1B, C, compare top and bottom panels). All stimuli were applied via a motion platform on which the head-fixed animal was placed (Fig. 1B, left). Notably, we compared the responses of the same vestibular thalamocortical and VO neural populations during both naturalistic and artificial stimulation.

### Vestibular thalamocortical neuronal responses represent instantaneous head velocity with markedly less ambiguity for naturalistic as compared to artificial stimulation

**Fig. 2:**
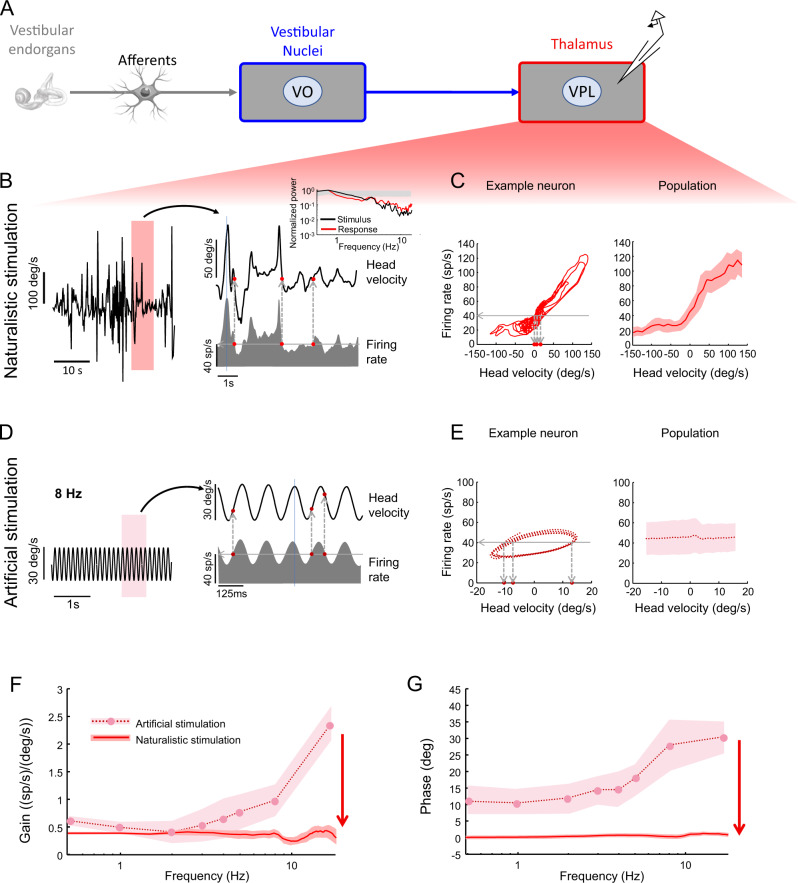
Vestibular thalamocortical neurons display reliable responses to naturalistic self-motion but not to artificial self-motion stimuli. **A** Schematic showing early and central vestibular pathways. Recordings were obtained from vestibular thalamocortical neurons. **B** *Left:* Time series showing a naturalistic self-motion stimulus. *Right:* A portion of this same stimulus corresponding to the rectangle (top) and the firing rate response (bottom) from the same vestibular thalamocortical neuron shown on the left. It is seen that the same firing rate (horizontal line) is primarily elicited by a smaller range of values of the head velocity, which leads to less ambiguity. *Inset:* Stimulus (black) and spike train (red) power spectra from this same neuron. The grey band shows the 95% confidence interval obtained from a Poisson process for which the power spectrum is independent of frequency. The center of the grey band is the power spectrum for a Poisson process whose firing rate is equal to the population-averaged firing rate of the data. **C** *Left:* Firing rate as a function of head velocity for the same example neuron shown in **B**. It is seen that the same firing rate (horizontal line) is primarily elicited by a smaller range of values of the head velocity, which leads to less ambiguity. *Right:* Population-averaged firing rate as a function of head velocity (*N* = 28). The band shows 1 SEM. **D** *Left:* Time series showing an artificial sinusoidal self-motion stimulus with frequency 8 Hz. *Right:* A portion of this same stimulus corresponding to the rectangle (top) and the firing rate response (bottom) from a typical vestibular thalamocortical neuron. It is seen that the same firing rate (horizontal line) can be elicited by multiple values of the head velocity (vertical lines), which leads to ambiguity. **E** *Left:* Firing rate as a function of head velocity for the same example neuron shown in B. It is seen that the same firing rate (horizontal dashed line) can be elicited by multiple values of the head velocity (vertical dashed lines), which leads to ambiguity. *Right:* Population-averaged firing rate as a function of head velocity (*N* = 28). The band shows 1 SEM. **F** Population-averaged neural gain as a function of frequency for artificial (dashed) and naturalistic (solid) self-motion (*N* = 28). The band shows 1 SEM. **G** Population-averaged phase as a function of frequency for artificial (dashed) and naturalistic (solid) self-motion (*N* = 28). The band shows 1 SEM.

We first focused on recordings obtained from vestibular thalamocortical neurons in response to naturalistic and artificial stimulation (Fig. 2A). The relationship between neural firing rate responses and the head velocity stimulus was characterized by computing gain and phase as a function of frequency using linear systems identification techniques (i.e., cross-spectral densities for naturalistic stimulation and cross-correlation functions for artificial stimulation; see Methods). Under naturalistic stimulation, we found that the time-dependent firing rate was well-aligned with the head velocity stimulus waveform with no inhibitory cutoff (Fig. 2B, right, compare black and grey). Indeed, a plot of an example neuron’s firing rate as a function of instantaneous head velocity revealed a strong correlation between both quantities (Fig. 2C, left). This is because a given firing rate (Fig. 2C, left, horizontal line) was consistently elicited by similar instantaneous head velocity values (Fig. 2C, left, vertical lines). Such robust encoding was confirmed independently as the response power spectrum strongly decayed with increasing frequency like that of the stimulus (Fig. 2B, inset). Qualitatively similar results were observed across our dataset when plotting the population-averaged firing rate as a function of instantaneous head velocity (Fig. 2C, right).

We then, for comparison, plotted the same example neuron’s firing rate as a function of instantaneous head velocity during artificial sinusoidal stimulation and found striking differences (Fig. 2D, E; example shown for 8 Hz). In contrast to results shown above for naturalistic stimulation (Fig. 2B, right), the firing rate response led (i.e., reached its maximum value before) the head velocity stimulus (Fig. 2D, right). As a result, a given firing rate (Fig. 2E, left, horizontal line) was elicited by different instantaneous head velocity values (Fig. 2E, left, vertical lines). Qualitatively similar results were observed across our dataset as shown in the right panel of Fig. 2E where the population-averaged firing rate is independent of instantaneous head velocity. Systematically varying sinusoidal frequency revealed that the fundamental difference observed in encoding naturalistic vs. artificial stimuli was robust across the natural frequency range. Specifically, during naturalistic stimulation, the firing rate response was well-aligned with the instantaneous head velocity stimulus as quantified by constant gain (Fig. 2F, solid) and no significant phase lead/lag (Fig. 2G, solid) for all frequencies. In contrast, during artificial stimulation, we found frequency-dependent response dynamics as the firing rate response instead encoded head velocity with gains (Fig. 2F, dashed) and significant phase leads (Fig. 2G, dashed) that both increased with increasing frequency. Thus, our results so far show that vestibular thalamocortical firing rate responses represent instantaneous head velocity quite differently during naturalistic as compared to artificial stimulation.

### Reduced coding ambiguity during naturalistic stimulation in vestibular thalamocortical neurons is not simply inherited from their vestibular nuclei input

**Fig. 3:**
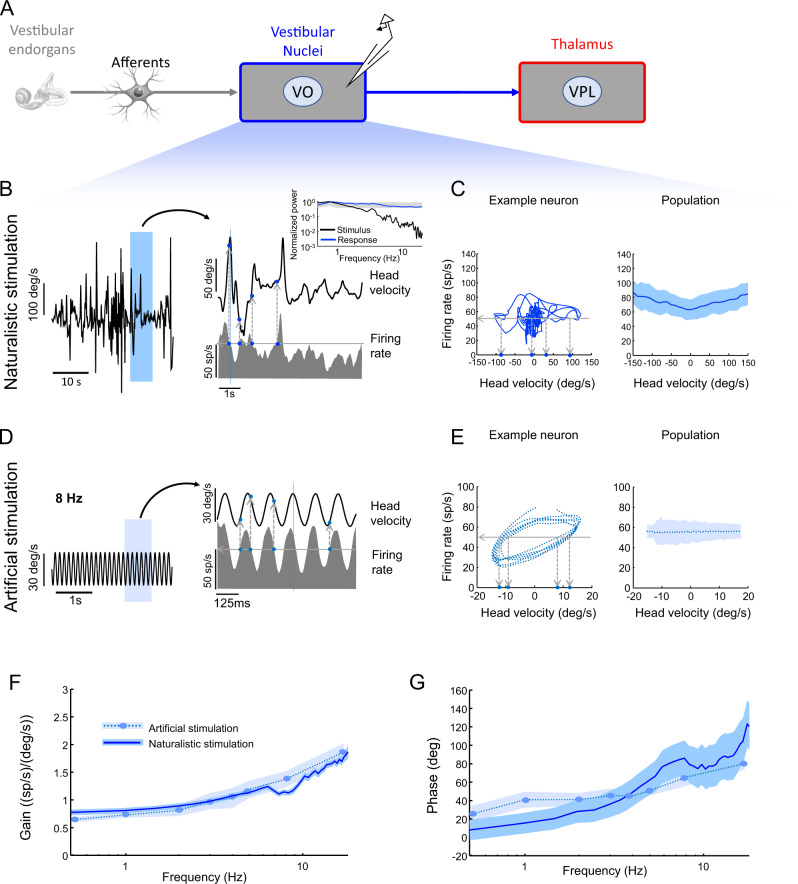
Changes in vestibular thalamocortical neural responses are not inherited from vestibular nuclei neurons. **A** Schematic showing early and central vestibular pathways and that recordings were obtained from VO neurons within the vestibular nuclei. **B** *Left:* Time series showing a naturalistic self-motion stimulus. *Right:* A portion of this same stimulus corresponding to the rectangle (top) and the firing rate response (bottom) from the same VO neuron shown on the left. It is seen that the same firing rate (horizontal line) can be elicited by multiple values of the head velocity (vertical dashed lines), which leads to similar ambiguity that is seen for artificial stimuli. *Inset:* Stimulus (black) and spike train (blue) power spectra from this same neuron. The grey band shows the 95% confidence interval obtained from a Poisson process for which the power spectrum is independent of frequency. The center of the grey band is the power spectrum for a Poisson process whose firing rate is equal to the population-averaged firing rate of the data. **C** *Left:* Firing rate as a function of head velocity for the same example neuron shown in B. It is seen that the same firing rate (horizontal line) can be elicited by multiple values of the head velocity (vertical dashed lines), which leads to similar ambiguity that is seen for artificial stimuli. *Right:* Population-averaged firing rate as a function of head velocity (*N* = 27). The band shows 1 SEM. **D** *Left:* Time series showing an artificial sinusoidal self-motion stimulus with frequency 8 Hz. *Right:* A portion of this same stimulus corresponding to the rectangle (top) and the firing rate response (bottom) from a typical VO neuron. It is seen that the same firing rate (horizontal line) can be elicited by multiple values of the head velocity (vertical dashed lines), which leads to ambiguity. **E** *Left:* Firing rate as a function of head velocity for the same example neuron shown in B. It is seen that the same firing rate (horizontal line) can be elicited by multiple values of the head velocity (vertical dashed lines), which leads to ambiguity. *Right:* Population-averaged firing rate as a function of head velocity (*N* = 27). The band shows 1 SEM. **F** Population-averaged neural gain as a function of frequency for artificial (dashed) and naturalistic (solid) self-motion (*N* = 27). The band shows 1 SEM. **G** Population-averaged phase as a function of frequency for artificial (dashed) and naturalistic (solid) self-motion (*N* = 27). The band shows 1 SEM.

Perhaps the simplest explanation for the experimentally observed differences in coding of instantaneous head velocity during naturalistic vs. artificial stimulation is that these are displayed by vestibular nuclei neurons and are then simply transmitted to vestibular thalamocortical neurons. To test this hypothesis, we performed the same analysis on the recorded activities of VO neurons within the vestibular nuclei during naturalistic and artificial stimulation (Fig. 3A). In contrast to vestibular thalamocortical neurons, we found that VO neurons similarly encoded instantaneous head velocity during both conditions. Specifically, the firing rate response led head velocity during both naturalistic (Fig. 3B) and artificial (Fig. 3D) stimulation. As a result, a given firing rate (left panels of Fig. 3C, E; horizontal lines) was elicited by different instantaneous head velocity values (left panels of Fig. 3C, E; vertical lines) in both cases. Qualitatively similar results were observed across our dataset when plotting the population-averaged firing rate as a function of instantaneous head velocity (right panels of Fig. 3C, E). Using the same approaches to estimate gain and phase above in Fig. 2 revealed that VO neurons displayed similar frequency-dependent response dynamics during both stimulation conditions that were characterized by gains (Fig. 3F, solid and dashed) and significant phase leads (Fig. 3G, solid and dashed) that increased in the same manner with increasing frequency. The similar frequency-dependent response dynamics resulted in comparable ambiguity values during both stimulation conditions.

**Fig. 4:**
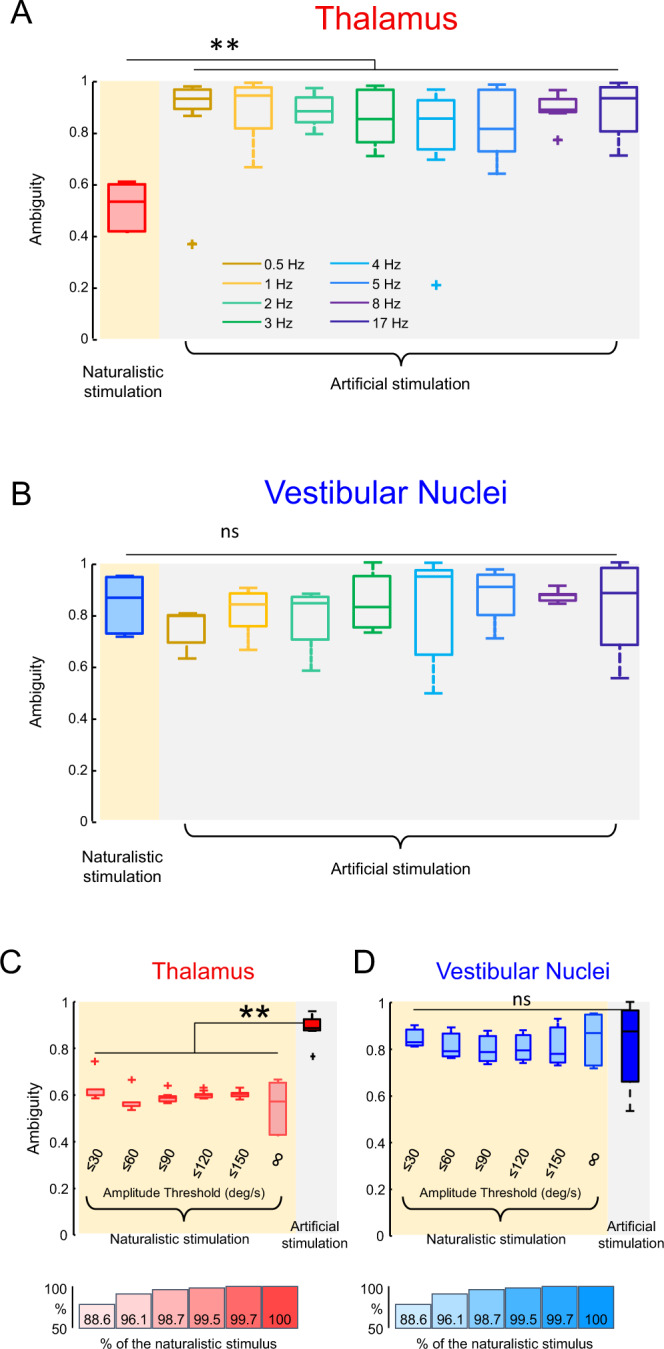
Vestibular thalamocortical but not vestibular nuclei neurons display significantly lower ambiguity during naturalistic stimulation as compared to artificial stimulation. **A** Whisker-box plots showing the ambiguity measure was significantly less for naturalistic (left) than artificial sinusoidal (right) stimuli for all frequencies tested (Kruskal-Wallis test, *N* = 28, 0.5 Hz: *p* = 8.97 × 10^−8^; 1 Hz: *p* = 8.98 × 10^−8^; 2 Hz: *p* = 1.85 × 10^−7^; 3 Hz: *p* = 6.72 × 10^−7^; 4 Hz: *p* = 4.22 × 10^−5^; 5 Hz: p = 1.24 × 10^−5^; 8 Hz: *p* = 9.06 × 10^−8^; 17 Hz: p = 8.97 × 10^−8^) for vestibular thalamocortical neurons. Individual datapoints indicate outliers. **B** Whisker-box plots showing the ambiguity measure was not significantly different for naturalistic (left) and artificial (right) stimuli for all frequencies tested (Kruskal-Wallis test, *N* = 27, 0.5 Hz: *p* = 0.039; 1 Hz: *p* = 0.997; 2 Hz: *p* = 0.706; 3 Hz: *p* = 1; 4 Hz: *p* = 0.999; 5 Hz: *p* = 0.963; 8 Hz: *p* = 0.984; 17 Hz: *p* = 1) for vestibular nuclei neurons. Individual datapoints indicate outliers. **C** Whisker-box plots showing population-averaged ambiguity measure values for vestibular thalamocortical neurons during naturalistic stimulation using different amplitude thresholds. All values were significantly lower than those obtained during artificial sinusoidal stimulation (Kruskal-Wallis test, *N* = 28, ≤30 deg/s: *p* = 0.003; ≤60 deg/s: *p* = 3.71 × 10^−8^; ≤90 deg/s: *p* = 3.71 × 10^−8^; ≤120 deg/s: *p* = 2.08 × 10^−7^; ≤150 deg/s: *p* = 6.52 × 10^−6^; ∞: *p* = 3.73 × 10^−8^). The percentage of the signal that is retained under each condition is denoted below. Specifically, ≤30 deg/s corresponds to 88.6%, ≤60 deg/s corresponds to 96.1%, ≤90 deg/s corresponds to 98.7%, ≤120 deg/s corresponds 99.5%, ≤150 deg/s corresponds to 99.7%. We note that the larger variance seen when considering the full amplitude range (“∞”) is most likely because there are fewer datapoints at large amplitudes, which results from the fact that the stimulus probability distribution is maximal around 0 deg/s. **D** Same as **C**, but for vestibular nuclei neurons. Ambiguity values obtained using different amplitude thresholds were not significantly different than those obtained during artificial stimulation (Kruskal-Wallis test, *N* = 27, ≤30 deg/s: *p* = 0.999; ≤60 deg/s: *p* = 0.856; ≤90 deg/s: *p* = 0.202; ≤120 deg/s: *p* = 0.392; ≤150 deg/s: *p* = 0.828; ∞: *p* = 0.999). Individual datapoints (“+”) indicate outliers. For all panels, whisker-box plots show the median, the lower and upper quartiles, any outliers (computed using the interquartile range), and the minimum and maximum values that are not outliers.

To quantify the fundamental difference in how vestibular thalamocortical neurons represent instantaneous head velocity during naturalistic vs. artificial stimulation, we computed an ambiguity measure ranging between 0 (no ambiguity) and 1 (almost complete ambiguity; see Methods). Overall, coding ambiguity values were significantly lower during naturalistic stimulation than during artificial stimulation irrespective of sinusoidal frequency (Fig. 4A, Kruskal-Wallis test, *p* ≤ 0.001 in all cases). Further, even after compensating for any phase differences between stimulus and response, we still observed significantly lower ambiguity values during naturalistic stimulation then during artificial stimulation (Supplementary Fig. 1, *p* ≤ 0.04 in all cases). In contrast, for vestibular nuclei neurons, ambiguity values obtained during naturalistic stimulation were not significantly different from those obtained during artificial stimulation irrespective of sinusoidal frequency (Fig. 4B, Kruskal-Wallis test, *p* ≥ 0.36 in all cases). Thus, taken together, these results demonstrate that differences in coding observed for vestibular thalamocortical neurons are not simply inherited from their vestibular nuclei input.

### Reduced coding ambiguity is not due to differences in amplitude between stimulation conditions

One important difference between the natural and artificial self-motion stimulation conditions is that, as mentioned above, the former reach higher amplitudes than the latter (Fig. 1B). To test whether differences in amplitude were responsible for the observed differences in coding, we first recorded the activities of vestibular thalamocortical neurons in response to artificial “ramp” stimuli consisting of a sinusoidal waveform whose amplitude increased linearly as a function of time (Supplementary Fig. 2). Overall, we found that response ambiguity did not decrease with increasing stimulus amplitude for all frequencies tested (Supplementary Figs. 2, 3, Wilcoxon rank sum test, *p* ≥ 0.27 in all cases). Second, we restricted the amplitude range of naturalistic stimuli and then computed ambiguity values for vestibular thalamocortical neurons (see Methods). Even for amplitudes as low as 30 deg/s, which are less than the maximum amplitude of ramp stimuli (100 deg/s), ambiguity values were still significantly lower than those obtained during artificial stimulation (Fig. 4C). As a control, the same analysis performed on vestibular nuclei neurons did not reduce ambiguity (Fig. 4D). Thus, taken together, these results provide strong evidence against the hypothesis that the observed differences in coding are due to differences in stimulus amplitude.

### Contrast gain control accounts for reduced coding ambiguity during naturalistic stimulation in vestibular thalamocortical neurons

**Fig. 5:**
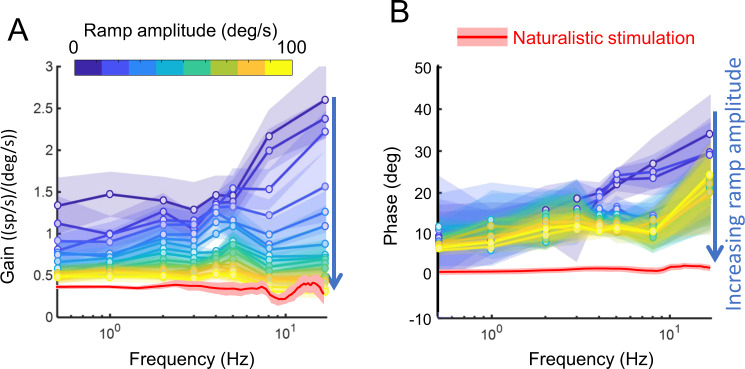
Vestibular thalamocortical neurons display nonlinear responses to stimuli with increasing amplitude. **A**, **B** Population-averaged gain **A** and phase **B** values as a function of frequency for different amplitude ranges (colorbar; *N* = 28). For comparison, population-averaged gain and phase values obtained for naturalistic self-motion stimuli are shown in red (*N* = 28). The bands show 1 SEM.

So far, our results have shown that vestibular thalamocortical neurons more reliably encode instantaneous head velocity during naturalistic than during artificial stimulation as quantified by reduced ambiguity. Additionally, our analysis above revealed that this property is neither simply inherited from vestibular nuclei nor due to differences in stimulus amplitude. Overall, the qualitatively different dependencies of gain and phase on frequency observed for vestibular thalamocortical neurons during naturalistic vs. artificial stimulation indicate that neural responses are nonlinear. To test whether such nonlinearities might account for reduced coding ambiguity during naturalistic stimulation, we first investigated whether vestibular thalamocortical neurons responded nonlinearly to the ramp stimuli described above. We found that neural gain decreased as a function of stimulus amplitude, while response phase was instead relatively independent of amplitude for a given frequency (Fig. 5A, B). Interestingly, response gain was independent of frequency for large ramp stimulus amplitudes, which is similar to what is observed under naturalistic stimulation (compare yellow and red curves in Fig. 5A). Notably, the observed decrease in gain with increasing amplitude is a hallmark of contrast gain control adaptation which constitutes, by definition, a nonlinearity^17–31^. Thus, we hypothesize that gain control adaptation accounts for reduced coding ambiguity during naturalistic stimulation in vestibular thalamocortical neurons.

**Fig. 6:**
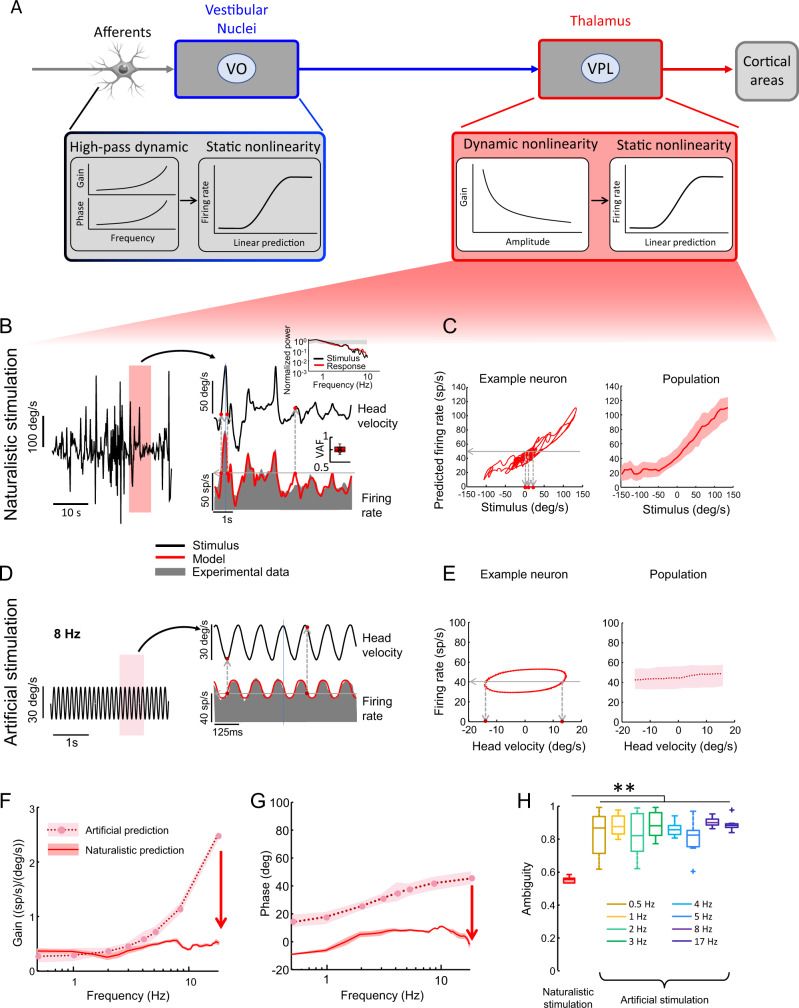
A mathematical model incorporating the known filtering properties of peripheral and early vestibular pathways and contrast gain control displayed by vestibular thalamocortical neurons can account for why naturalistic but not artificial self-motion stimuli are reliably encoded with reduced ambiguity. **A** Schematic showing early and central vestibular pathways (top) and the corresponding components of our model (bottom). We focus on the vestibular thalamocortical component here. **B** Naturalistic self-motion stimulus timeseries (black), corresponding experimentally obtained neural firing rate response from an example neuron (grey), and model prediction (red). It is seen that a lesser range of head velocity values (vertical dashed lines) give rise to the same firing rate (horizontal dashed line), which leads to less ambiguity. *Inset*: Population-averaged variance-accounted-for (VAF, *N* = 28). The whisker-box plot shows the median, the lower and upper quartiles, any outliers (computed using the interquartile range), and the minimum and maximum values that are not outliers. **C** *Left:* Predicted firing rate as a function of head velocity. It is seen that a lesser range of head velocity values (vertical dashed lines) give rise to the same firing rate (horizontal line), which leads to less ambiguity. *Right:* Population-averaged predicted firing rate as a function of head velocity. The band shows 1 SEM. **D** Artificial sinusoidal stimulus time series (black), corresponding experimentally obtained neural firing rate response (grey), and model prediction (red). **E** *Left:* Predicted firing rate as a function of head velocity. It is seen that a greater range of head velocity values (vertical dashed lines) give rise to the same firing rate (horizontal line), which leads to more ambiguity. *Right:* Population-averaged predicted firing rate as a function of head velocity (*N* = 28). The band shows 1 SEM. **F** Population-averaged predicted neural gain as a function of frequency for artificial (dashed) and naturalistic (solid) self-motion (*N* = 28). The band shows 1 SEM. **G** Population-averaged predicted phase as a function of frequency for artificial (dashed) and naturalistic (solid) self-motion (*N* = 28). The band shows 1 SEM. **H** Population-averaged ambiguity values from our model were significantly less for naturalistic (left) than for artificial (right) stimuli for all frequencies tested (Kruskal-Wallis test, *N* = 28, 0.5 Hz: *p* = 3.94 × 10^−7^; 1 Hz: *p* = 8.97 × 10^−8^; 2 Hz: *p* = 9.28 × 10^−8^; 3 Hz: *p* = 8.97 × 10^−8^; 4 Hz: *p* = 6.46 × 10^−7^; 5 Hz: *p* = 0.005; 8 Hz: *p* = 8.97 × 10^−8^; 17 Hz: *p* = 8.97 × 10^−8^). Individual datapoints indicate outliers. Each whisker-box plot shows the median, the lower and upper quartiles, any outliers (computed using the interquartile range), and the minimum and maximum values that are not outliers.

To test this hypothesis, we built a mathematical model (see Methods and Fig. 6A) that incorporated the frequency-dependent dynamics of vestibular nuclei neurons as well as the observed contrast gain control of vestibular thalamocortical neurons for different frequencies (Supplementary Fig. 4). Static nonlinearities were also included to account for known rectification and saturation properties. We first fit our model to experimental data recorded from each vestibular thalamocortical neuron during ramp stimulation (Supplementary Fig. 5). We then compared the firing rate predicted by our model to that observed experimentally during naturalistic stimulation (Fig. 6B). Overall, we found an excellent match between experimentally observed and predicted firing rate responses (Fig. 6B, compare red and grey), as quantified by large variance-accounted-for (VAF; Fig. 6B, bottom inset). Notably, consistent with experimental observations (Fig. 2B, C), the predicted firing rate response was well-aligned with the head velocity stimulus waveform (Fig. 6B, compare black and red traces). As a result, there was a strong positive correlation between both quantities (Fig. 6C). Likewise, during artificial stimulation, we found an excellent match between experimentally observed and predicted firing rate responses (Fig. 6D, compare red and grey). Specifically, again consistent with experimental observations (Fig. 2D, E), the model firing rate response led the head velocity stimulus (Fig. 6D). This significant phase lead gave rise to a weaker correlation between both quantities (Fig. 6E). Applying linear systems identification further revealed that our vestibular thalamocortical neuron model successfully reproduced the experimentally observed qualitative differences in neural gain (Fig. 6F) and phase (Fig. 6G) during naturalistic and artificial stimulation (compare with Fig. 2F, G, respectively). Thus, once again consistent with experimental observations (Fig. 4A), ambiguity values computed from our model were consistently significantly lower for the naturalistic as compared to the artificial stimulation condition (Fig. 6H).

Taken together, these results show that our vestibular thalamocortical neuron model incorporating contrast gain control adaptation can successfully reproduce the coding differences seen experimentally during naturalistic and artificial stimulation. In contrast, our vestibular nuclei neuron model, which did not incorporate contrast gain control adaptation, displayed similar frequency-dependent response dynamics during both stimulation conditions (Supplementary Fig. 6) as seen experimentally (compare with Fig. 3). The key distinction between our vestibular nuclei and thalamocortical neuron models is that the former does not include contrast gain control adaptation. Thus, our modeling results demonstrate that contrast gain control adaptation accounts for reduced coding ambiguity during naturalistic stimulation in vestibular thalamocortical neurons.

### Vestibular thalamocortical neurons more optimally encode naturalistic self-motion stimuli than vestibular nuclei neurons irrespective of sensory representation

**Fig. 7:**
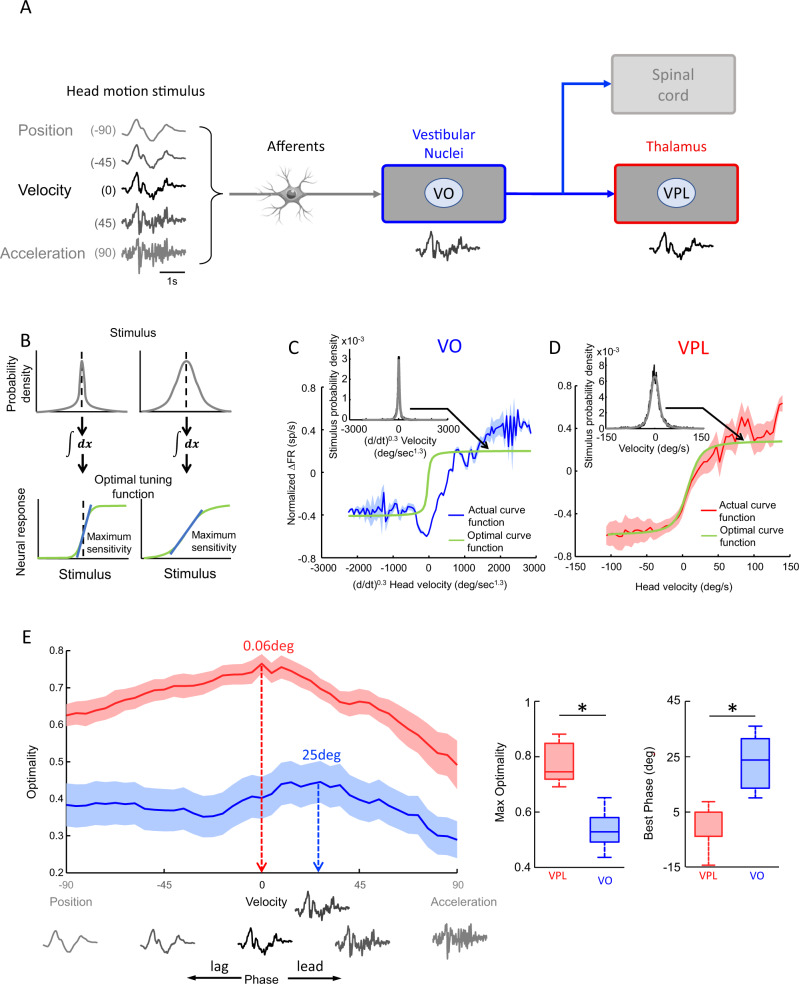
Vestibular thalamocortical neurons more optimally encode natural vestibular stimuli than vestibular nuclei neurons. **A** Schematic showing a snippet of a naturalistic self-motion stimulus (left) represented in terms of going from position (top) to acceleration (bottom). Afferents synapse onto VO neurons within the vestibular nuclei (blue) that project to vestibular thalamocortical neurons within the thalamus (red) as well as to the spinal cord (grey). **B** Illustration of how the neural tuning curve must match stimulus statistics in order to coding to be optimized. Shown are two example stimulus probability densities (top) and the resulting optimal tuning functions (bottom, green) obtained by integrating them. Also indicated is the fact that maximum sensitivity (blue lines) occurs for stimulus values that are most likely to occur. **C** Population-averaged actual (blue) and optimal (green) tuning functions for VO neurons based on the stimulus probability distribution (inset). In the inset: black is the data and grey is a Gaussian fit (*N* = 27). The band shows 1 SEM. **D** Same as **C**, but for vestibular thalamocortical neurons (*N* = 28). The band shows 1 SEM. **E** *Left:* Population-averaged optimality values for vestibular thalamocortical (*N* = 28, red) and VO (*N* = 27, blue) neurons as a function of the stimulus feature going from position to acceleration. *Right:* Population-averaged maximum optimality values were significantly higher for vestibular thalamocortical (*N* = 28) than for VO (*N* = 27) neurons (top, two-sided Wilcoxon rank sum test, *p* = *0.01*). Best phase was significantly greater for VO *N*(=27) than for vestibular thalamocortical (*N* = 28) neurons (bottom, two-sided Wilcoxon rank sum test, *p* = *0.03*). The bands show 1 SEM. Each whisker-box plot shows the median, the lower and upper quartiles, any outliers (computed using the interquartile range), and the minimum and maximum values that are not outliers.

We next investigated if the responses of vestibular thalamocortical neurons were more optimized (i.e., transmit more information) than those of vestibular nuclei neurons at encoding naturalistic self-motion stimuli (Fig. 7A). On the one hand, it could be argued that ambiguous encoding by vestibular nuclei neurons can be resolved by considering a transformation of the head velocity stimulus (i.e., a sensory representation) that is in phase with the firing rate response. For example, self-motion can be represented not only in terms of time-varying head velocity, but also in terms of time-varying head position, head acceleration, or any waveform in between. If this is the case, then vestibular thalamocortical neural responses would not necessarily be more optimized than those of vestibular nuclei neurons. On the other hand, if vestibular thalamocortical neurons display contrast gain control adaptation, then their responses to naturalistic stimuli should be more optimized independent of sensory representation.

Thus, to address whether vestibular thalamocortical neural responses to naturalistic self-motion stimuli are more optimized than those of vestibular nuclei neurons, we considered sensory representations of the stimulus obtained by either integrating or differentiating the time-varying head velocity waveform. Specifically, integration gives rise to position with a 90° phase lag relative to velocity, differentiation gives rise to acceleration with a 90° phase lead relative to velocity, while partial integration or differentiation will give rise to phase lags and leads between these two phases, respectively (Fig. 7A, left). Optimality of coding was then tested by comparing the neural tuning curve (i.e., the relationship between the neural firing rate response and the input) obtained experimentally to that predicted from optimal coding theory by integrating the input probability distribution (Fig. 7B).

Figure 7C, D shows the population-averaged optimal (green) and actual (blue, red) tuning curves for VO and vestibular thalamocortical neurons during naturalistic stimulation, respectively. Specifically, we identified the sensory representation that was best aligned to the firing rate response by partially differentiating the head velocity stimulus, thereby minimizing the phase difference between the two and reducing coding ambiguity (see Methods). For VO neurons, we found that there was a significant mismatch between the actual and optimal tuning curves, which is indicative of suboptimal coding (Fig. 7C, compare blue and green). In contrast, for vestibular thalamocortical neurons, we found an excellent match between the actual and optimal tuning curves (Fig. 7D, compare red and green). Importantly, coding optimality was systematically greater for vestibular thalamocortical neurons as compared to VO neurons irrespective of sensory representation (Fig. 7E, left). Thus, our results demonstrate that reduced ambiguity due to contrast gain control adaptation in vestibular thalamocortical neurons does not compromise coding efficiency. Comparable analysis applied to artificial sinusoidal stimuli revealed markedly different results; optimality values obtained for VO and vestibular thalamocortical neurons were comparable and both were significantly lower than those obtained during naturalistic stimulation, which indicates that coding of artificial stimuli is suboptimal (Supplementary Fig. 7).

Finally, we computed the sensory representation for which optimality of coding was maximal during naturalistic stimulation for both VO and vestibular thalamocortical neurons. We found that, while vestibular thalamocortical neurons were most efficient at encoding head velocity, vestibular nuclei neurons were most efficient at encoding a sensory representation between velocity and acceleration (Fig. 7E, compare red and blue). This is consistent with the prominent phase leads observed for the latter but not the former in response to naturalistic stimulation (Figs. 3G and 2G, respectively). The implications of this result are further considered below in the discussion.

## Discussion

We investigated the function of sensory adaptation in the encoding of naturalistic stimuli in the vestibular system. In response to naturalistic stimuli, vestibular thalamocortical neurons encoded instantaneous head velocity with significantly less ambiguity than their input vestibular nuclei neurons. In contrast, in response to artificial stimuli, neurons in both brain areas displayed similar frequency-dependent response dynamics characterized by significant phase leads that led to an ambiguous relationship between the firing rate response and the instantaneous head velocity stimulus. Further analysis of responses to stimuli with increasing amplitude revealed a strong decrease in gain for vestibular thalamocortical neurons, which is indicative of contrast gain control adaptation. Using computational methods, we demonstrated that such adaptation accounts for the differences in coding that were observed experimentally under naturalistic vs. artificial stimulation. Finally, comparison of actual tuning curves vs. those predicted from optimal coding theory revealed that vestibular thalamocortical neurons consistently displayed greater optimality of coding than vestibular nuclei neurons irrespective of sensory representation. Thus, taken together, our results show that sensory adaptation reduces ambiguity without compromising optimality. As such, our study challenges the common wisdom that optimal coding by sensory adaptation comes at the cost of increased ambiguity. Rather, we propose that sensory adaptation underlies unambiguous optimized encoding of natural stimuli.

Our results show that vestibular thalamocortical neurons more optimally encode natural self-motion than vestibular nuclei neurons irrespective of sensory representation (e.g., head velocity, acceleration, position, etc…) (Fig. 7E). However, information is only useful to an organism if decoded by downstream brain areas. Vestibular thalamocortical neurons project to vestibular cortical neurons within areas such as the parieto-insular vestibular cortex (PIVC), the ventral intraparietal area (VIP), area 2 v of the intraparietal sulcus, and area 3a in the sulcus centralis^43–46^. How these vestibular cortical neurons respond to naturalistic self-motion stimuli such as those used in this study remains unknown to date since prior studies have focused on artificial stimulation^40,47–50^. We predict that vestibular cortical neurons will even more optimally encode naturalistic self-motion stimuli than vestibular thalamocortical neurons. We further speculate that vestibular cortical neurons provide an unambiguous encoding of head velocity to give rise to perception. Interestingly, the fact that previous studies have shown that head velocity detection thresholds (i.e., the minimum stimulus amplitude that can be perceived) are approximately constant for frequencies above 0.5 Hz^51,52^ supports our prediction that perception is centered on head velocity rather than another sensory representation (e.g., acceleration). Nevertheless, further investigation focusing on how vestibular cortical neurons respond to natural self-motion stimuli is necessary. Additionally, it is important to consider that natural stimuli experienced during everyday life comprise both active and passive self-motion. Previous studies have shown that the vestibular thalamocortical neurons considered here display markedly attenuated responses to active self-motion consisting of head and/or body orienting movements^40^. Similar results were obtained for VO neurons within the vestibular nuclei (see^34,53^ for review). Thus, further studies will be needed to establish how both vestibular thalamocortical and VO neurons encode natural active self-motion signals (e.g., those experienced during locomotion).

Our results show that, in response to naturalistic stimulation, VO neurons within the vestibular nuclei instead best encoded a sensory representation between head velocity and acceleration. This is because these neurons displayed gains and phase leads that both increased as a function of frequency. This raises two questions: (1) why do VO neurons display such frequency-dependent response dynamics to naturalistic stimuli and; (2) why are these response dynamics effectively cancelled in the thalamus? In answer to the first question, it is important to note that the frequency-dependent response dynamics displayed by VO neurons are largely inherited from the periphery. Specifically, vestibular nuclei neurons and afferents with more irregular resting discharge (i.e., irregular afferents) demonstrate similar frequency-dependent response dynamics to head rotations^37,54^. This is consistent with electrophysiological findings showing that VO neurons are more likely to receive input from irregular afferents^55,56^. In answer to the second question, prior studies have shown that VO neurons do not only mediate self-motion perception but also contribute to the control of posture. Specifically, VO neurons project not only to the thalamus but also to the spinal cord^55,56^, thus mediating vestibulo-spinal reflexes. Such reflexes require compensating the dynamic load of the head-neck system and it has been proposed that irregular afferents, with their increased gains and phase leads as compared to regular afferents, are best suited to achieve this function^57,58^ (see^34^ for review). Accordingly, the frequency-dependent response dynamics displayed by irregular afferents and their target VO neurons are most likely needed to compensate for the inertia of the head-neck system when generating vestibulo-spinal reflexes.

Our finding that contrast gain control in vestibular thalamocortical neurons mediates reliable encoding of natural stimuli leads to the question of what mechanisms underlie this adaptation. Contrast gain control adaptation has been observed ubiquitously across systems and species^17–31^ (see^12^ for review). For example, in the visual system, contrast gain control has been shown to occur in several areas including the retina, lateral geniculate nucleus of the thalamus^59^, and visual cortex^28,60,61^. Contrast gain control has also been observed in the auditory^18^ and somatosensory cortices^24,62,63^. Previous studies conducted in other systems have proposed that various mechanisms underlie contrast gain control adaptation including synaptic depression^27^, intrinsic connections^28^, and cortico-thalamic feedback^29^ (but see^21^). Theoretically, all of these factors could contribute to the observed contrast gain control adaptation in vestibular thalamocortical neurons. For example, previous anatomical studies have identified various types of local inhibitory interneurons intrinsic to VPL^64,65^. Further electrophysiological studies are however needed to test whether these interneurons can provide the necessary population normalizing drive. Moreover, area VPL of the thalamus is known to receive feedback from cortical areas^66^. Contrast gain control could also be due to synaptic depression at VN-VPL synapses. While such synaptic depression has been observed at equivalent synapses other areas of the thalamus (e.g., retina-LGN)^30,31^, it remains unknown if VN-VPL synapses display comparable depression. Further studies are needed to uncover the nature of the mechanism(s) leading to the observed contrast gain control adaptation in vestibular thalamocortical neurons.

Our results showing that contrast gain control adaptation reduces ambiguity and thus permits a reliable representation of the current head velocity are surprising. This is because the prevailing view is that contrast gain control serves to optimize the coding of stimuli with changing statistics^12^ at the expense of increased ambiguity^23,32,33^. For example, in the rodent somatosensory system, adaptation provides increased information about stimulus history but increases ambiguity about the current stimulus value when using artificial stimuli^32^. Our results using artificial self-motion stimuli are consistent with this view as coding ambiguity values approached their maxima. Conversely, when using naturalistic stimuli, we found significantly reduced coding ambiguity in vestibular thalamocortical neurons. Computational approaches further show that contrast gain control adaptation can account for this result. Thus, an important question is: how does such adaptation permit reliable encoding? To answer this question, it is interesting to note that natural stimuli are typically characterized by a time-varying waveform whose amplitude (i.e., envelope) varies slowly^2–4,6,67^. In particular, the amplitude of natural self-motion stimuli (i.e., the natural self-motion envelope) varies relatively slowly with spectral frequency content below 2 Hz^68^. We speculate that it is this particular feature of natural self-motion stimuli that is necessary to reduce coding ambiguity. Specifically, studies performed in other systems have shown that contrast gain control adaptation can occur relatively fast with timescales of 100 msec or less^18^, which is significantly lower than the timescale of natural self-motion envelopes (∼500 msec). We predict that contrast gain control adaptation in vestibular thalamocortical neurons occurs within a comparable timeframe (e.g., 100 msec). If this is the case, given that the envelope timescale is much greater, then the responses of vestibular thalamocortical neurons would be well-aligned with the stimulus waveform. Moreover, optimality of coding would not be compromised as there would be a match between the neural tuning curve and current stimulus statistics (e.g., head velocity probability density). Further studies using artificial stimuli whose amplitude varies more quickly in time (e.g., step increases in stimulus amplitude such as those considered by^23^) are needed to verify this prediction.

Finally, we speculate that contrast gain control adaptation will mediate reliable encoding of natural stimuli in other sensory systems for which the adaptation timescale is much shorter than that of the envelope. This appears to be the case in the visual system where the timescale of adaptation^69^ is much shorter than that of natural stimuli^70^ (see^3^ for review). Further studies are needed to test this prediction.

## Methods

### Ethics

All experimental procedures were approved by the McGill University and Johns Hopkins University Animal Care Committees. Procedures were in compliance with the guidelines of the Canadian Council on Animal Care. Recording experiments were conducted in three rhesus monkeys (2 male, ages 8 and 10 years, 7.2 and 8 kg, and 1 female aged 10 years, 10.1 kg). The animals were housed in pairs on a 12 h light/dark cycle. They were brought to the laboratory, about three times a week, for approximately two hours recording sessions. s. All animals had participated in previous experiments in our laboratory but all of them were in good health condition and did not require any medication.

### Surgical procedures

Two male and one female rhesus monkeys (*Macaca Mulatta*) were prepared for acute extracellular recordings using aseptic MRI guided surgical techniques^40^. Surgical levels of isoflurane (0.8–1.5%) were maintained during surgery during which the animals were implanted with a custom-made medical grade titanium head post for restraining the head and a recording chamber that was placed based on the co-registration of a CT scan, an MRI scan and the rhesus brain atlas in Brainsight (Brainsight 2 Vet, Rogue Research, Montreal, Canada) such as to provide access to VPL. VPL access position was confirmed post-surgery by the co-registration of a second CT scan with a recording electrode maintained in the center of the recording chamber. The implant was chronically fastened to the skull with titanium screws and Simplex P bone cement (Stryker Orthopedics, Mahwah, NJ). An 18 mm eye coil (3 loops of Teflon-insulated stainless steel wire) was also implanted behind the conjunctiva of one eye in each monkey^71^. Finally, buprenorphine (0.01 mg/kg, IM) and cefazolin (25 mg/kg) were administered as postoperative analgesia and antibiotic, respectively. Animals recovered for at least 2 weeks before recordings began.

### Data acquisition

Throughout recordings, head-restrained monkeys were seated in a primate chair that was mounted on a motion platform rotating about the vertical axis (i.e., yaw rotation) within a dark room. Eye movements were measured using the magnetic search coil technique^71^. Turntable velocity was controlled by REX, a QNX based, real-time data acquisition system^72^, and measured using an angular rate sensor (Watson Industries, Inc., Eau Claire, WI, USA). All behavioral signals were low-pass filtered at 250 Hz, and acquired at 1 kHz sampling frequency. The VPL was located relative to the lateral geniculate nucleus, which was recognizable due to the presence of individual neurons that responded to either the onset or offset of a light flashed while lowering the electrode during early recordings^73^. Each neuron included in the present report demonstrated robust firing rate modulation during sinusoidal, whole-body rotations, and no gain to eye movements during saccades or smooth pursuit^42,74^. Further, we ensured that neurons did not respond to visual stimulation caused by small spots of light presented in the visual field^73^. Extracellular single-unit activity was recorded using enamel-insulated tungsten microelectrodes (2–10 MΩ impedance, Frederick Haer, Bowdoin, ME, USA), band-pass filtered from 300 Hz to 3 kHz, and sampled at 30 kHz. Both neural and behavioral data were acquired through the Cerebus Neural Signal Processor (Blackrock Microsystems, Salt Lake City, UT, USA).

### Stimulation

Neurons were initially identified on the basis of their response to passive rotations in the dark in the absence of visual stimulation (i.e., whole-body rotations). The same vestibular stimuli were then applied with the lights on to confirm that there was no change in mean firing rate or gain due to responses to visual stimulation. In addition, we confirmed that all neurons did not respond to horizontal or vertical eye movements. Our self-motion stimuli consisted of sinusoidal whole-body rotations along the yaw axis with frequencies 0.5, 1, 2, 3, 4, 5, 8, and 17 Hz whose amplitude was either constant (15 deg/s) or increased linearly between 0 and 100 deg/s within a duration of 100 s (ramp-up) and then back to 0 deg/s within a duration of 100 s (ramp-down). Overall, variations in neural activity were similar during both the ramp-up and ramp-down portions of the stimulus. Results were thus averaged between both portions. Finally, we used naturalistic stimuli whose timecourse closely mimicked that of signals recorded while the animal performed natural behaviors (e.g., walking, jumping, etc…)^36,37^. All animals were awake and behaving during stimulation.

### Data analysis

Neural data was imported into Matlab (The Mathworks, Natick, MA, USA) for sorting as well as for all offline analysis. For each neuron, spike times were converted into a binary sequence sampled at 1 kHz. Specifically, time was discretized into bins of 1 ms length and the content of each bin was set to 1000 if one spike occurred within and to 0 otherwise. The time-dependent firing rate was obtained by low-pass filtering the binary sequence with a Kaiser filter whose cutoff was 0.5 Hz above the stimulus frequency^75^. We recorded neurons that demonstrated excitatory responses for ipsilaterally and contralaterally-directed rotations (i.e., type I vs. type II neurons, respectively). We recorded from 18 type I and 10 type II neurons in VPL and 16 type I and 11 type II VO neurons within the vestibular nuclei. For type II neurons, the head velocity stimulus was inverted (i.e., multiplied by −1) as there were otherwise no differences between the activities of these neurons and those of type I neurons, as done previously^37,76^.

For sinusoidal stimuli, the firing rate was aligned to be in phase with the head velocity stimulus by computing the cross-correlation and finding the position of the peak, as done previously^54^. The phase was then obtained from this lag. The gain was computed from the slope of the best-fit straight line when plotting the firing rate aligned with the head velocity stimulus. For naturalistic stimuli, the gain and phase were computed from the cross-spectrum between the head velocity and the firing rate divided by the stimulus power spectrum, as done previously^37^. For ramp stimuli, the firing rate during each time window was aligned to be in phase with the head velocity stimulus by computing the cross-correlation and finding the position of the peak, as done previously^54^. The phase was then obtained from this lag. The gain was computed from the slope of the best-fit straight line when plotting the firing rate aligned with the head velocity stimulus. In all cases, ambiguity was computed as 1-|R | , where R is the Pearson correlation coefficient between the firing rate response and the head velocity stimulus. This is because our results show that the firing rate response of vestibular thalamocortical neurons is well-aligned with the head velocity stimulus waveform as quantified by negligible phase differences. Thus, if there is no significant correlation between firing rate and instantaneous head velocity (e.g., Fig. 2E), then the ambiguity measure will be close to unity (i.e., almost complete ambiguity). In contrast, with increasing correlation between firing rate and instantaneous head velocity (e.g., Fig. 2C), the ambiguity measure approaches zero (i.e., less ambiguity). We note that computing ambiguity using instead a Spearman correlation coefficient gave rise to qualitatively similar results. Ambiguity values were also computed between the head velocity stimulus and the firing rate after it was aligned to be in phase by computing the cross-correlation and finding the peak as described above. For some analyses (Fig. 4), we used an amplitude threshold to identify epochs of the naturalistic stimulus which we included in the analysis to compute the ambiguity measure. Specifically, we only considered stimulus and firing rate response segments for which the stimulus amplitude was lower or equal to the threshold value. While this method creates gaps in the head velocity and firing rate time series that are then concatenated, this is not an issue for our data since our results show that there is no significant phase difference between head velocity and firing rate response for vestibular thalamocortical neurons during naturalistic stimulation when the full continuous head velocity signal is considered. We further note that, because the stimulus probability distribution is maximal at 0 deg/s, over 85% of the time series are retained even for the lowest threshold value considered (Fig. 4).

Sensory representations of the head velocity stimulus were obtained by applying a fractional differentiation operator $\frac{{d}^{\alpha }}{{{dt}}^{\alpha }}$ to the head velocity time-varying waveform^77^. Here α is the order and ranges between −1 and 1 (note that negative values of α correspond to integration). In the frequency domain, fractional differentiation of order α corresponds to filtering by a transfer function *H*_*α*_ (*f*) given by^78^:

$${H}_{\alpha }\left(f\right)={\left(2\pi f\right)}^{\alpha }\,{{\exp }}\left(\frac{i\alpha \pi }{2}\right)$$

with $i=\sqrt{-1}$. For the example VO neuron shown in Fig. 7C, the order of the derivative was set such that the phase difference between the resulting sensory representation and the firing rate response was minimized as assessed by computing the cross-correlation function between both signals and finding the lag at which the maximum value is attained. Optimal tuning functions were obtained by integrating the probability distribution of the sensory representation^8^ and then compared to experimentally obtained tuning function computed by plotting the firing rate as a function of the sensory representation (Fig. 7). This same analysis was also applied to artificial sinusoidal stimuli (Supplementary Fig. 6).

### Modeling

Our mathematical modeling included both the high pass filtering properties of vestibular nuclei neurons as well as the contrast gain control observed for vestibular thalamocortical neurons. High pass filtering properties were modeled using the following transfer function:

1 $$T\left(f\right)={{{\rm{k}}}}\frac{s\left(s{{{{\rm{T}}}}}_{1}+1\right)}{\left(s\,{{{{\rm{T}}}}}_{2}+1\right)\left(s\,{{{{\rm{T}}}}}_{{{{\rm{c}}}}}+1\right)}$$

where *s* = 2*πif*, $i=\sqrt{-1}$, k = 1.7207 spk/s/(deg/s), T_1_ = 0.06 s, T_2_ = 0.0006 s, T_c_ = 5.7 s are time constants representing the dynamics of sensory transduction and afferent filtering properties^79,80^.

Contrast gain control was assumed to be instantaneous such that the time-varying gain *G*(*t*) was given as a Lorentzian function of the time-varying amplitude *A*(*t*):

2 $$G\left(t\right)=\frac{{{{{\rm{P}}}}}_{1}}{{{{{\rm{P}}}}}_{2}A\left(t\right)+1}$$

where P_1_, P_2_ are parameters that were obtained by fitting the function to a plot of the gain as a function of stimulus amplitude obtained from the data. We used P_1_ = 2 spk/s/(deg/s) and P_2_ = 0.037 s/deg. The time-varying amplitude was obtained from the absolute value of the Hilbert transform of the head velocity stimulus *H*(*S*(*t*)), which allows us to write the stimulus *S*(*t*) as:

3 $$S\left(t\right)=A\left(t\right)\,{{\cos }}\left(2\pi f\left(t\right)\,t\right)$$

where the amplitude *A*(*t*) is given by |*H*(S(*t*))*| and* the time varying frequency *f*(*t*) is given by:

4 $$f\left(t\right)={{\arctan }}\left(\frac{{{{\rm{imag}}}}\left(\right. H\left(S\left(t\right)\right)}{{{{\rm{real}}}}\left(\right.H\left(S\left(t\right)\right)}\right)/(2\pi t)$$

Contrast gain control was implemented by multiplying the time-varying stimulus *S*(*t*) by the time-varying gain *G*(*t*). We note that this differs from other implementations of contrast gain control (e.g., Naka-Rushton) used to characterize the relationship between the stimulus amplitude and the neural response instead of explicitly considering changes in neural gain^81^.

The static nonlinearity of vestibular nuclei neurons was assumed to be a sigmoidal function:

5 $${{{\rm{SIG}}}}\left(X\right)=-25+\frac{50}{1+{{\exp }}\left[-\frac{\left(X\right)}{10}\right]}$$

which is consistent with that obtained in previous studies^36^. Finally, as done in previous studies^36,80^, the form of the static nonlinearity of vestibular thalamocortical neurons was obtained by plotting the predicted output of the model before the static nonlinearity as a function of the actual firing rate and then discretizing the x-axis using 100 bins to obtain the mean value. The dependency was then quantified using by fitting a 6^th^ order polynomial to the data. The final output of the model was then given by applying this function to the predicted output of the model before the static nonlinearity. Agreement between modeling and experimental data was quantified using the variance-accounted-for (VAF), which is computed as:

6 $${{{\rm{VAF}}}}=1-\frac{{{{\rm{var}}}}\left({{{{\rm{FR}}}}}_{{{{\rm{pred}}}}}-{{{{\rm{FR}}}}}_{{{{\rm{actual}}}}}\right)}{{{{\rm{var}}}}\left({{{{\rm{FR}}}}}_{{{{\rm{actual}}}}}\right)}$$

where FR_pred_ is the firing rate predicted from the model, FR_actual_ is the experimentally obtained firing rate, and var(…) is the variance.

### Statistics

MATLAB (The MathWorks, Natick MA) was used for statistical analysis. N represents the number of cells analyzed in each paradigm. Our sample sizes were similar to those generally employed in the field^36,37,82^. Before statistical analysis, normality of distribution was evaluated using a Shapiro-Wilk’s test. All data were tested for the presence of non-stationarities using an augmented Dickey-Fuller test. We did not find any significant non-stationarities during either resting discharge or driven activities for either of afferents or central vestibular neurons (*p* > 0.05 in all cases). To determine if variances between groups were comparable, an F-test was used. Statistical significance (*p* < 0.05) was determined using parametric analysis with either two-tailed Student’s *t* test or one-way ANOVA. Post hoc pairwise comparisons were conducted using Tukey’s honestly significant difference test or Dunnett’s test. Throughout, values are expressed as mean ± SEM.

### Reporting summary

Further information on research design is available in the Nature Research Reporting Summary linked to this article.

## Supplementary information

Supplementary Information

Reporting Summary

## Data Availability

The data generated in this study have been deposited in the Zenodo database 10.5281/zenodo.6381003^83^. Source data are provided in this paper.

## Source data

Source Data

## Code availability

The codes used in this study have been deposited in the Zenodo database 10.5281/zenodo.6381003^83^.
