# Supplementary material for: Sensory adaptation mediates efficient and unambiguous encoding of natural stimuli by vestibular thalamocortical pathways
Source: Nat Commun. 2022 May 12;13:2612. doi: 10.1038/s41467-022-30348-x (PMC9098492; doi:10.1038/s41467-022-30348-x)
Supplement: Supplementary file 1 — Supplementary Information [file 41467_2022_30348_MOESM1_ESM.pdf]

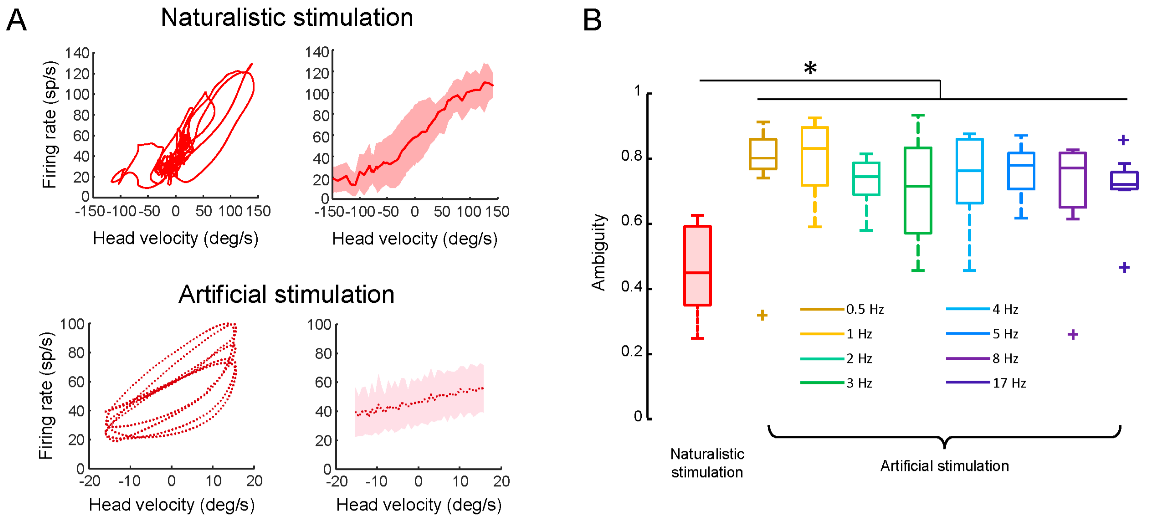

Supplementary Figure 1. *Compensating for phase differences between the head velocity stimulus and firing rate response does not affect the qualitative nature of our results.* **A)** *Left:* firing rate as a function of head velocity for an example vestibular thalamocortical neuron after compensating for phase differences between stimulus and response during naturalistic (top) and artificial (bottom) stimulation. The band shows 1 SEM. *Right:* Population-averaged firing rate as a function of head velocity after compensating for phase differences between stimulus and response during naturalistic (top) and artificial (bottom) stimulation (N=28). The band shows 1 SEM. **B)** Whisker-box plots showing the ambiguity measure computed after compensating for phase differences between stimulus and response was significantly less for naturalistic (left) than artificial sinusoidal (right) stimuli for all frequencies tested (Kruskal-Wallis test, N=28; 0.5 Hz: $p=8.97\times10^{-8}$; 1 Hz: $p=8.97\times10^{-8}$; 2 Hz: $p=3.3\times10^{-5}$; 3 Hz: p=0.0001; 4 Hz: $p=5.94\times10^{-7}$; 5 Hz: $p=9.72\times10^{-8}$; 8 Hz: $p=1.33\times10^{-5}$; 17 Hz: $p=4.91\times10^{-5}$). These plots show the median, the lower and upper quartiles, any outliers (computed using the interquartile range), and the minimum and maximum values that are not outliers.

Supplementary figure 2

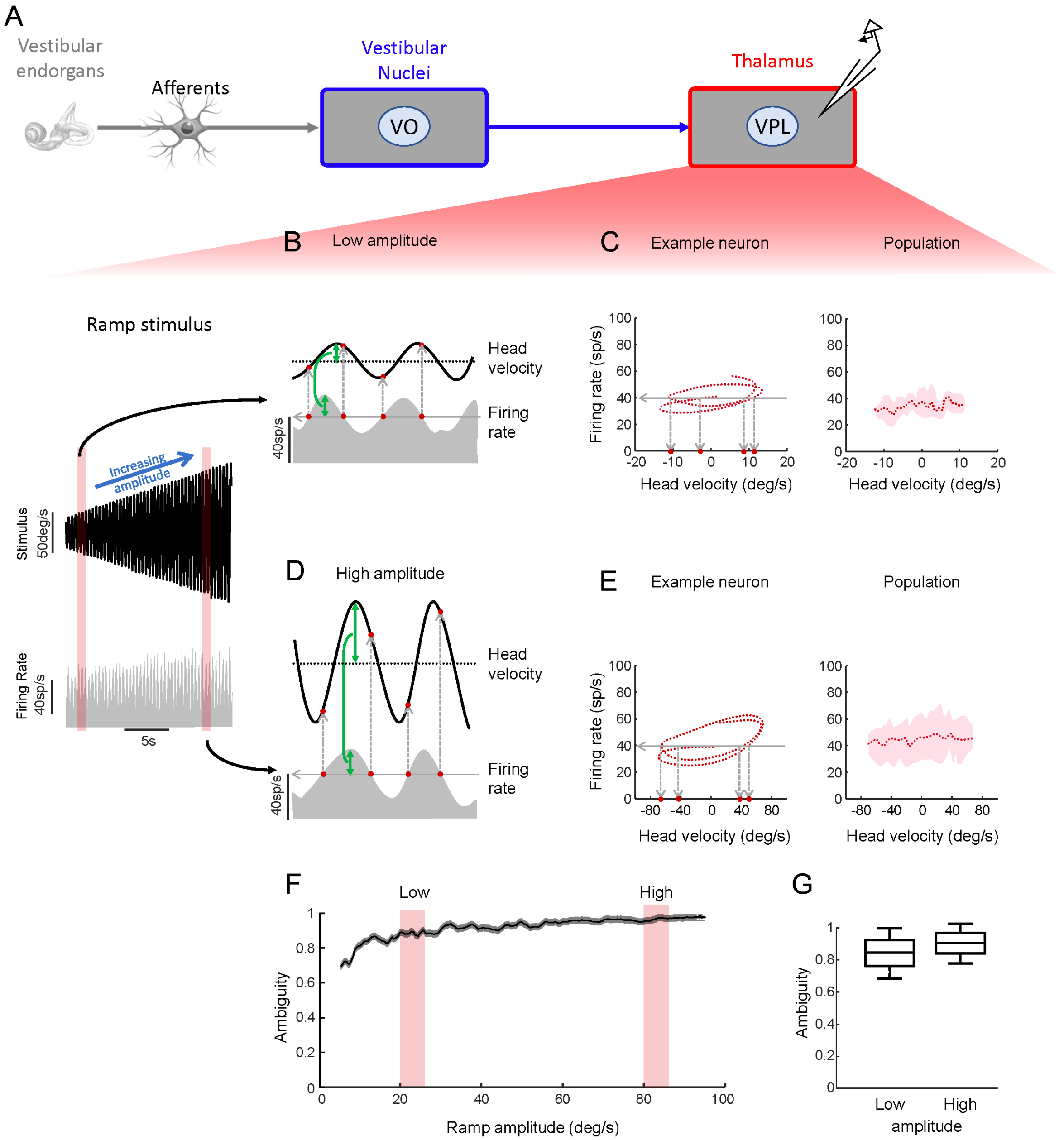

Supplementary Figure 2. *Response ambiguity of vestibular thalamocortical neurons does not decrease with increasing stimulus amplitude.* **A)** Schematic showing early and central vestibular pathways and that recordings were obtained from vestibular thalamocortical neurons. **B)** *Left:* Time series showing an artificial ramp stimulus consisting of a 2 Hz sinusoidal self-motion stimulus whose amplitude increases linearly with time (top, black) and the corresponding firing rate response from a typical vestibular thalamocortical neuron (bottom, gray). *Right:* A portion of this same stimulus corresponding to the rectangle (top, black) for a low amplitude and the firing rate response (bottom, gray) from a typical vestibular thalamocortical neuron. It is seen that the same firing rate (horizontal line) can be elicited by multiple values of the head velocity (vertical dashed lines), which leads to ambiguity. **C)** *Left:* Firing rate as a function of head velocity for the same example neuron shown in B. It is seen that the same firing rate (horizontal line) can be elicited by multiple values of the head velocity (vertical dashed lines), which leads to ambiguity. *Right:* Population-averaged firing rate as a function of head velocity (N=28). The band shows 1 SEM. **D)** Same as B, except for a high amplitude portion of the ramp stimulus and corresponding neural response (N=28). **E)** Same as C, except for a high amplitude portion of the ramp stimulus and corresponding neural response (N=28). The band shows 1 SEM. **F)** Population-averaged ambiguity as a function of stimulus amplitude (N=28). The band shows 1 SEM. **G)** Whisker-box plots showing population-averaged ambiguity values for low (left) and high (right) amplitudes (N=28). These plots show the median, the lower and upper quartiles, any outliers (computed using the interquartile range), and the minimum and maximum values that are not outliers.

Supplementary figure 3

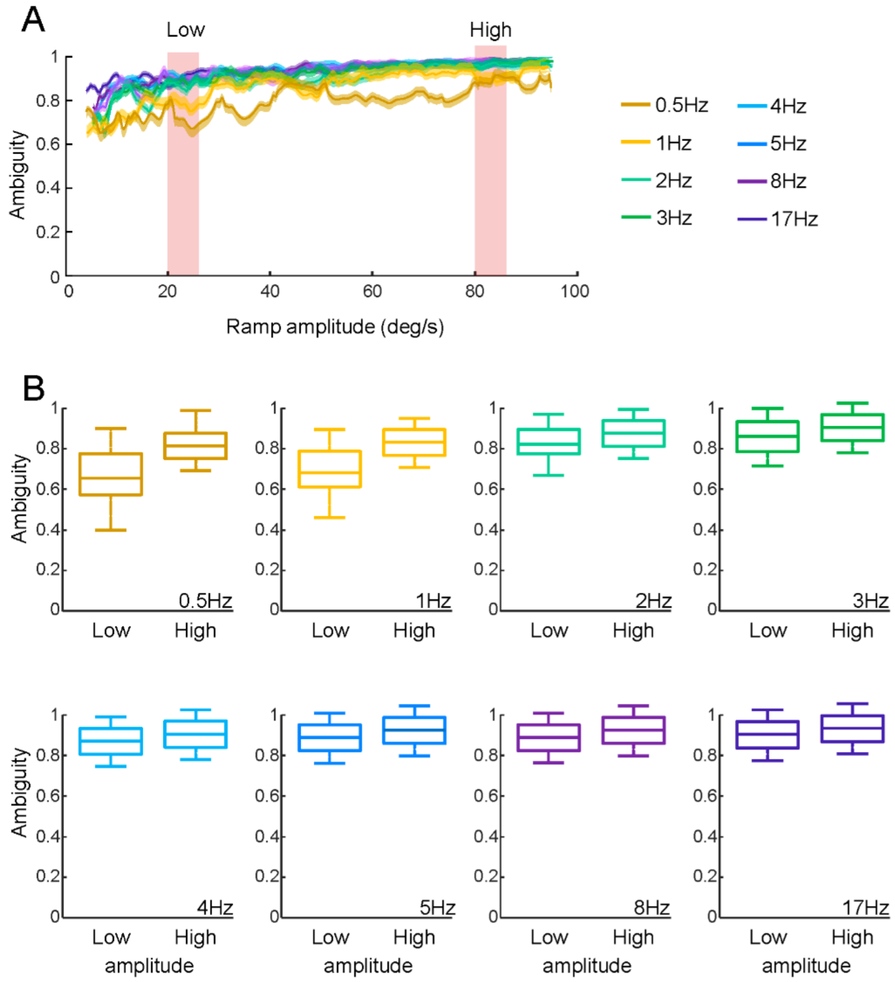

Supplementary Figure 3. *Response ambiguity of vestibular thalamocortical neurons does not decrease with increasing stimulus amplitude for all frequencies tested.* **A)** Population-averaged ambiguity as a function of stimulus amplitude for all sinusoidal stimuli (N=28). The bands show 1 SEM. **B)** Whisker-box plots showing population-averaged ambiguity values for low (left) and high (right) amplitudes for each frequency. No significant difference was observed for all frequencies tested (two-sided Wilcoxon rank sum tests, N=28; 0.5 Hz: p=0.17; 1 Hz: p=0.17; 2 Hz: p=0.18; 3 Hz: p=0.08; 4 Hz: p=0.13; 5 Hz: p=0.08; 8

Hz: p=0.07; 17 Hz: p=0.13). These plots show the median, the lower and upper quartiles, any outliers (computed using the interquartile range), and the minimum and maximum values that are not outliers.

Supplementary figure 4

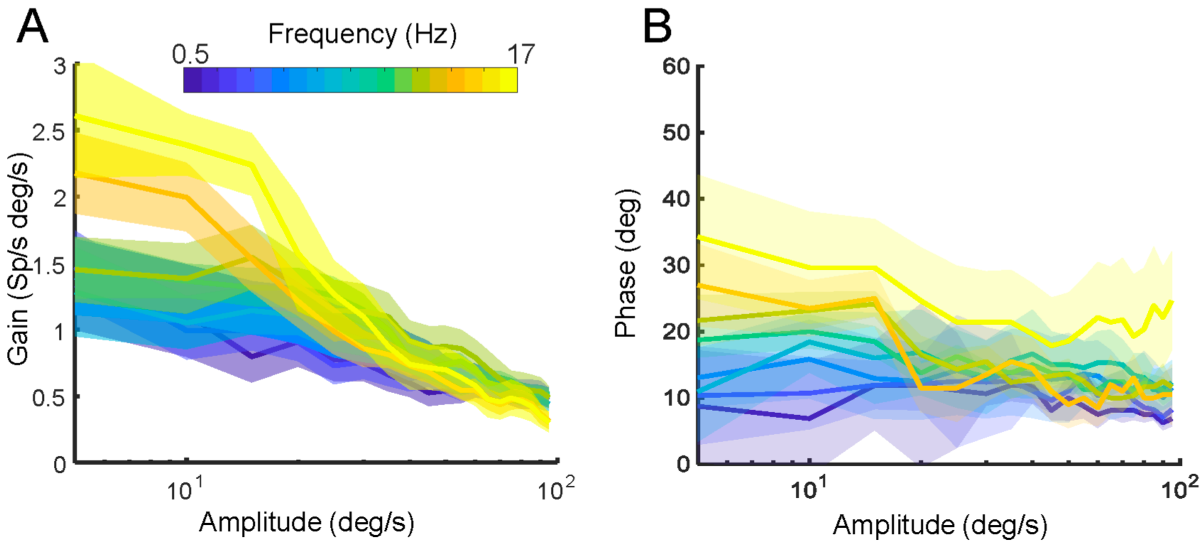

Supplementary Figure 4. *Vestibular thalamocortical neurons display nonlinear responses to ramp stimuli.* **A,B)** Population-averaged gain (A) and phase (B) values as a function of amplitude (N=28). The bands show 1 SEM.

Supplementary figure 5

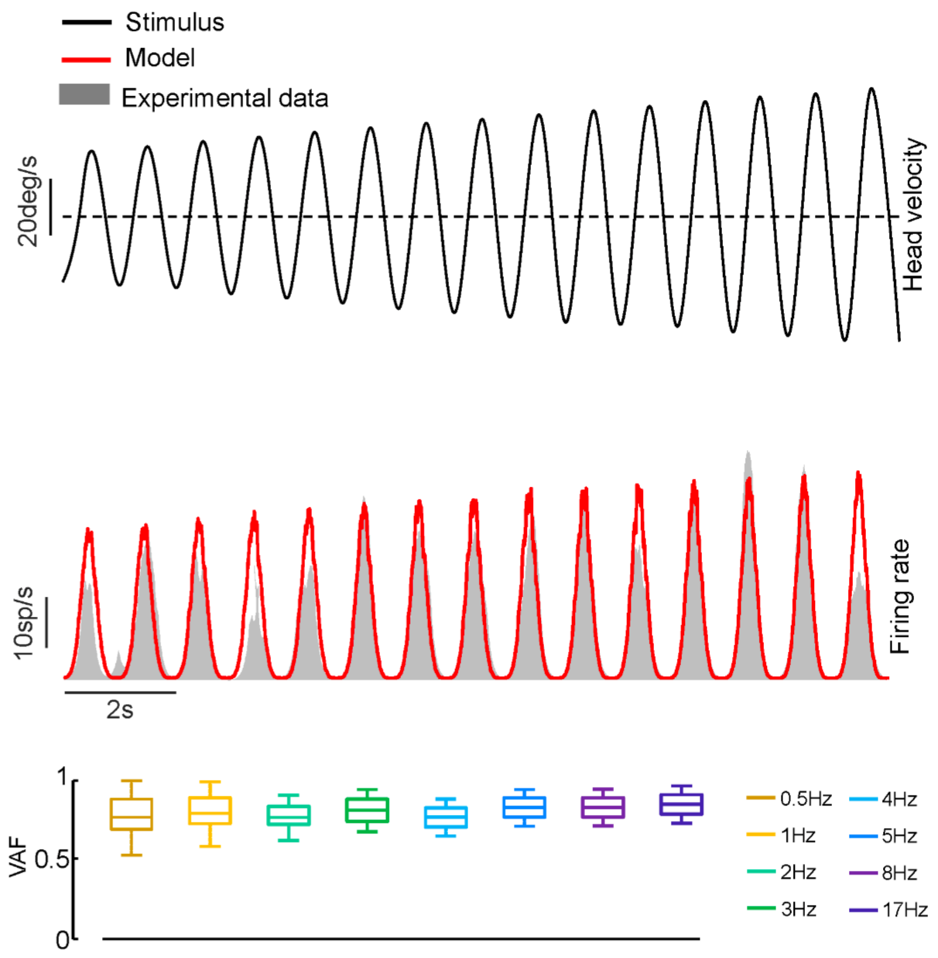

Supplementary Figure 5. Ramp stimulus (top) as well as neural response (bottom, gray) and model prediction (red, bottom). *Inset:* whisker-box plots Population-averaged VAF values obtained from fitting the model to experimental data for all sinusoidal artificial ramp stimulus frequencies (N=28). These plots show the median, the lower and upper quartiles, any outliers (computed using the interquartile range), and the minimum and maximum values that are not outliers.

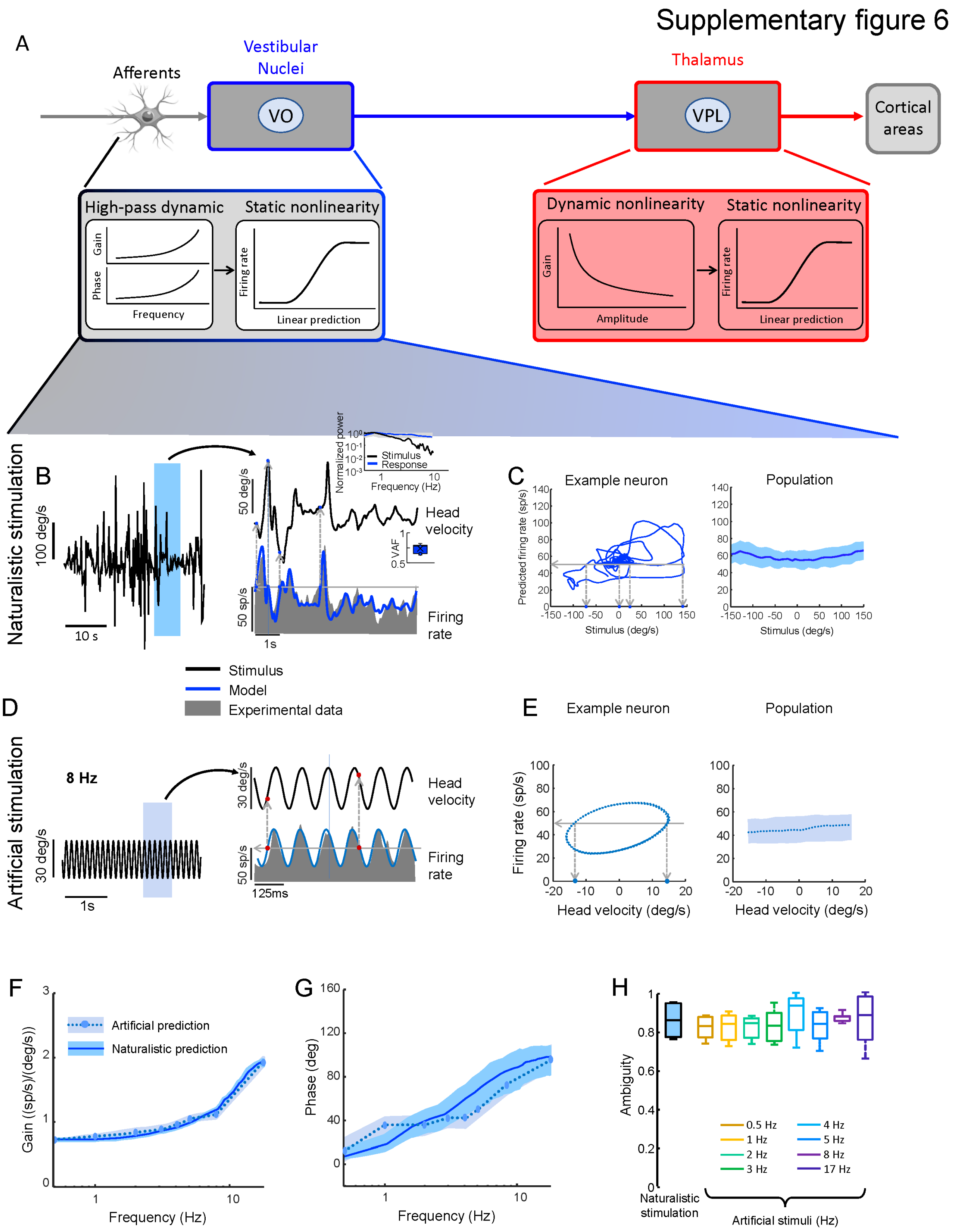

Supplementary Figure 6. *A mathematical model incorporating the known filtering properties of peripheral and early vestibular pathways can explain why vestibular nuclei neurons respond similarly to artificial and naturalistic self-motion stimuli.* **A)** Schematic showing early and central vestibular pathways (top) and the corresponding components of our model (bottom). We focus on the afferent-vestibular nuclei component here. **B)** Naturalistic self-motion stimulus timeseries (black), corresponding experimentally obtained

neural firing rate response from an example neuron (gray), and model prediction (blue). It is seen that multiple values of the head velocity (vertical dashed lines) give rise to the same firing rate (horizontal line), which leads to ambiguity. *Inset*: Population-averaged variance-accounted-for (VAF, N=27). **C)** *Left:* Predicted firing rate as a function of head velocity. It is seen that multiple values of the head velocity (vertical dashed lines) give rise to the same firing rate (horizontal line), which leads to ambiguity. *Right:* Population-averaged predicted firing rate as a function of head velocity (N=27). The band shows 1 SEM. **D)** Artificial sinusoidal stimulus time series (black), corresponding experimentally obtained neural firing rate response (gray), and model prediction (blue). **E)** *Left:* Predicted firing rate as a function of head velocity. It is seen that multiple values of the head velocity (vertical dashed lines) give rise to the same firing rate (horizontal line), which leads to ambiguity. *Right:* Population-averaged predicted firing rate as a function of head velocity (N=27). The band shows 1 SEM. **F)** Population-averaged predicted neural gain as a function of frequency for artificial (dashed) and naturalistic (solid) self-motion (N=27). The band shows 1 SEM. **G)** Population-averaged predicted phase as a function of frequency for artificial (dashed) and naturalistic (solid) self-motion (N=27). The band shows 1 SEM. **H)** Population-averaged predicted ambiguity values obtained from our model were not significantly different between naturalistic stimulation (left) and artificial sinusoidal stimulation for all frequencies tested (Kruskal-Wallis test, N=27, $p \geq 0.31$ in all cases). Each whisker-box plot shows the median, the lower and upper quartiles, any outliers (computed using the interquartile range), and the minimum and maximum values that are not outliers.

Supplementary figure 7

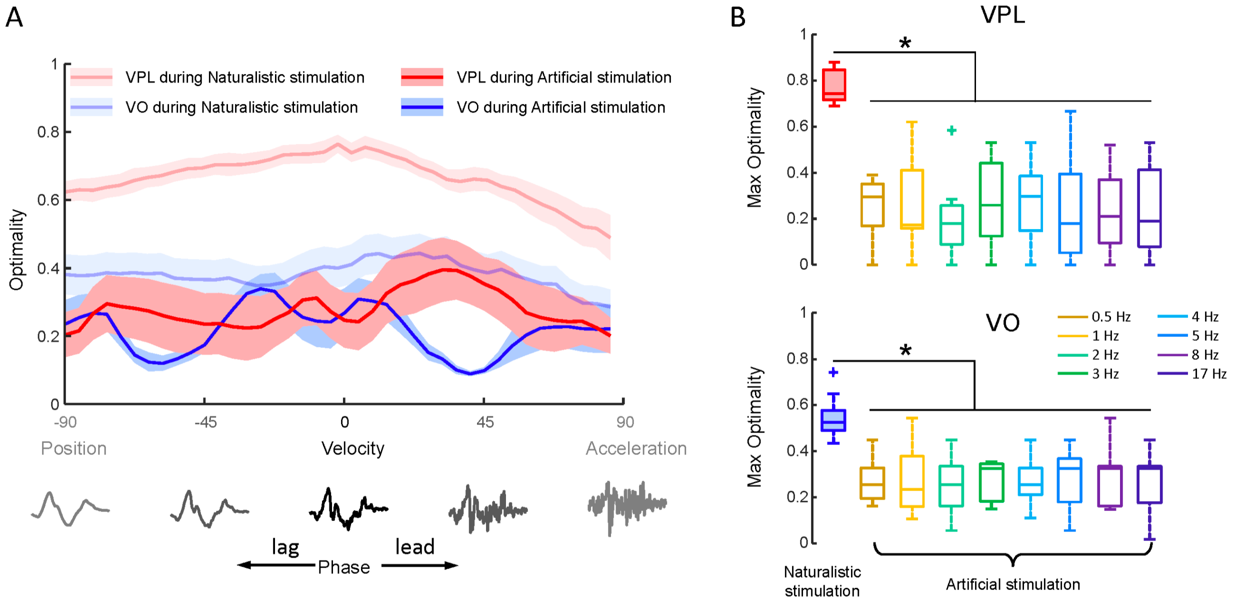

Supplementary Figure 7. *Naturalistic stimuli are more optimally encoded than artificial stimuli by both vestibular thalamocortical and VO neurons.* **A)** Population-averaged optimality values for vestibular thalamocortical (N=28, red) and VO (N=27, blue) neurons as a function of the stimulus feature going from position to acceleration during artificial sinusoidal stimulus with 8 Hz frequency were similar. Also shown in shaded colors are the curves from Fig. 5E obtained during naturalistic stimulation for comparison. The bands show 1 SEM. **B)** Population-averaged maximum optimality values were significantly higher during naturalistic than during artificial stimulation for all frequencies tested (VPL, N=28: Kruskal-Wallis, 0.5 Hz: p=1.93×10^{-7}; 1 Hz: p=2.32×10^{-7}; 2 Hz: p=8.97×10^{-8}; 3 Hz: p=4.07×10^{-7}; 4 Hz: p=2.02×10^{-6}; 5 Hz: p=8.98×10^{-8}; 8 Hz: p=9.52×10^{-8}; 17 Hz: p=9.52×10^{-8}; VO, N=27: Kruskal-Wallis, 0.5 Hz: p=1.05×10^{-7}; 1 Hz: p=9.73×10^{-8}; 2 Hz: p=8.97×10^{-8}; 3 Hz: p=1.51×10^{-7}; 4 Hz: p=9.11×10^{-8}; 5 Hz: p=4.51×10^{-7}; 8 Hz: p=2.03×10^{-7}; 17 Hz: p=1.05×10^{-7}). Each whisker-box plot shows the median, the lower and upper quartiles, any outliers (computed using the interquartile range), and the minimum and maximum values that are not outliers.
