# Supplementary material for: Sensory adaptation mediates efficient and unambiguous encoding of natural stimuli by vestibular thalamocortical pathways
Source: Nat Commun. 2022 May 12;13:2612. doi: 10.1038/s41467-022-30348-x (PMC9098492; doi:10.1038/s41467-022-30348-x)
Supplement: Supplementary file 2 — Reporting Summary [file 41467_2022_30348_MOESM2_ESM.pdf]

nature portfolio

Corresponding author(s): Maurice Chacron

Last updated by author(s): Jul 21, 2021

Reporting Summary

Nature Portfolio wishes to improve the reproducibility of the work that we publish. This form provides structure for consistency and transparency in reporting. For further information on Nature Portfolio policies, see our Editorial Policies and the Editorial Policy Checklist.

Statistics

For all statistical analyses, confirm that the following items are present in the figure legend, table legend, main text, or Methods section.

n/a	Confirmed	
☐	☒	The exact sample size (*n*) for each experimental group/condition, given as a discrete number and unit of measurement
☒	☐	A statement on whether measurements were taken from distinct samples or whether the same sample was measured repeatedly
☐	☒	The statistical test(s) used AND whether they are one- or two-sided<br>*Only common tests should be described solely by name; describe more complex techniques in the Methods section.*
☒	☐	A description of all covariates tested
☐	☒	A description of any assumptions or corrections, such as tests of normality and adjustment for multiple comparisons
☐	☒	A full description of the statistical parameters including central tendency (e.g. means) or other basic estimates (e.g. regression coefficient) AND variation (e.g. standard deviation) or associated estimates of uncertainty (e.g. confidence intervals)
☐	☒	For null hypothesis testing, the test statistic (e.g. *F*, *t*, *r*) with confidence intervals, effect sizes, degrees of freedom and *P* value noted<br>*Give P values as exact values whenever suitable.*
☒	☐	For Bayesian analysis, information on the choice of priors and Markov chain Monte Carlo settings
☒	☐	For hierarchical and complex designs, identification of the appropriate level for tests and full reporting of outcomes
☐	☒	Estimates of effect sizes (e.g. Cohen's *d*, Pearson's *r*), indicating how they were calculated

Our web collection on statistics for biologists contains articles on many of the points above.

Software and code

Policy information about availability of computer code

Data collection	Single unit activity, gaze and head signals were collected with Cerebus Neural Signal Processor (Blackrock)
Data analysis	After recordings, off-line analyses of neural data were performed using custom algorithms (MATLAB 2019, The MathWork). The codes have been deposited in the Zenodo database https://doi.org/10.5281/zenodo.6381003.

For manuscripts utilizing custom algorithms or software that are central to the research but not yet described in published literature, software must be made available to editors and reviewers. We strongly encourage code deposition in a community repository (e.g. GitHub). See the Nature Portfolio guidelines for submitting code & software for further information.

Data

Policy information about availability of data

All manuscripts must include a data availability statement. This statement should provide the following information, where applicable:

- Accession codes, unique identifiers, or web links for publicly available datasets
- A description of any restrictions on data availability
- For clinical datasets or third party data, please ensure that the statement adheres to our policy

The data generated in this study have been deposited in the Zenodo database https://doi.org/10.5281/zenodo.6381003.

Field-specific reporting

Please select the one below that is the best fit for your research. If you are not sure, read the appropriate sections before making your selection.

☒ Life sciences ☐ Behavioural & social sciences ☐ Ecological, evolutionary & environmental sciences

For a reference copy of the document with all sections, see nature.com/documents/nr-reporting-summary-flat.pdf

Life sciences study design

All studies must disclose on these points even when the disclosure is negative.

Sample size	Sample size comprise 55 neurons (28 VTN, 27 VO). The sample sizes are comparable to those used by previous studies in the field.
Data exclusions	Neurons were excluded if the isolation was lost before the end of the protocol.
Replication	All neurons were recorded from independently and were characterized using standard methods. Analyses on the population showed reproducibility of the results.
Randomization	Randomization is not applicable as the neuron type was determined based on its physiology.
Blinding	Blinding is not relevant for our study because the neuronal response cannot be influenced by the experimenter.

Reporting for specific materials, systems and methods

We require information from authors about some types of materials, experimental systems and methods used in many studies. Here, indicate whether each material, system or method listed is relevant to your study. If you are not sure if a list item applies to your research, read the appropriate section before selecting a response.

Materials & experimental systems

n/a	Involved in the study	
☒	☐	Antibodies
☒	☐	Eukaryotic cell lines
☒	☐	Palaeontology and archaeology
☐	☒	Animals and other organisms
☒	☐	Human research participants
☒	☐	Clinical data
☒	☐	Dual use research of concern

Methods

n/a	Involved in the study	
☒	☐	ChIP-seq
☒	☐	Flow cytometry
☒	☐	MRI-based neuroimaging

Animals and other organisms

Policy information about studies involving animals; ARRIVE guidelines recommended for reporting animal research

Laboratory animals	2 male rhesus monkeys ages 8 and 10 years, 7.2 and 8 kg, and 1 female aged 10 years, 10.1 kg
Wild animals	The study did not involve wild animals.
Field-collected samples	The study did not involve samples collected in the field.
Ethics oversight	All experimental protocols were approved by McGill and Johns Hopkins's University Animal Care Committees and were in compliance with the guidelines of the Canadian Council on Animal Care and of the NIH.

Note that full information on the approval of the study protocol must also be provided in the manuscript.
